# An Optimized Maximum Second-Order Cyclostationary Blind Deconvolution and Bidirectional Long Short-Term Memory Network Model for Rolling Bearing Fault Diagnosis

**DOI:** 10.3390/s25051495

**Published:** 2025-02-28

**Authors:** Jixin Liu, Liwei Deng, Yue Cao, Chenglin Wen, Zhihuan Song, Mei Liu, Xiaowei Cui

**Affiliations:** 1 School of Automation, Guangdong University of Petrochemical Technology, Maoming 525000, China; liujixin2000@gdupt.edu.cn (J.L.); dengliwei5603@yeah.net (L.D.); caoyue2000@gdupt.edu.cn (Y.C.); wencl@gdupt.edu.cn (C.W.); songzhihuan@gdupt.edu.cn (Z.S.); liumei@gdupt.edu.cn (M.L.); 2 School of Information and Control Engineering, Jilin Institute of Chemical Technology, Jilin 132022, China; 3 School of Computer Science, Baicheng Normal University, Baicheng 137000, China

**Keywords:** fault diagnosis, maximum second-order cyclostationarity blind deconvolution (CYCBD), bidirectional long short-term memory (BiLSTM), golden jackal optimization (GJO)

## Abstract

To address the challenge of extracting fault features and accurately identifying bearing fault conditions under strong noisy environments, a rolling bearing failure diagnostic technique is presented that utilizes parameter-optimized maximum second-order cyclostationary blind deconvolution (CYCBD) and bidirectional long short-term memory (BiLSTM) networks. Initially, an adaptive golden jackal optimization (GJO) algorithm is employed to refine important CYCBD parameters. Subsequently, the rolling bearing failure signals are filtered and denoised using the optimized CYCBD, producing a denoised signal. Ultimately, the noise-reduced signal is fed into the BiLSTM model to realize the classification of faults. The experimental findings demonstrate the suggested approach’s strong noise reduction performance and high diagnostic accuracy. The optimized CYCBD–BiLSTM improves the accuracy by approximately 9.89% compared with other methods when the signal-to-noise ratio (SNR) reaches −9 dB, and it can be effectively used for diagnosing rolling bearing faults under noisy backgrounds.

## 1. Introduction

As industrial development progresses, mechanical equipment becomes more complex and sophisticated. Rolling bearings, critical components of such machinery, frequently operate at high speed and in heavy load conditions, where damage is inevitable. This damage can significantly affect the machinery’s performance and lead to severe accidents [1]. Researchers are increasingly interested in monitoring data through sensors to ensure the proper mechanical device operation [2,3]. Accurate rolling bearing condition diagnosis is essential to preserving equipment functionality and preventing financial losses and safety risks from bearing failure [4,5].

In the realm of industrial production, rolling bearings often operate in complex environments. This complexity means that fault signals extracted from these environments typically contain considerable noise, necessitating preprocessing of the gathered vibration data. A pivotal development in this field is represented by the technique known as empirical mode decomposition (EMD) [6]. EMD works by breaking down the input into intrinsic mode functions (IMFs) to distinguish noise from the core signal. Nevertheless, EMD is prone to issues like mode mixing and end effects. In response, various EMD-inspired signal processing methodologies have emerged, including ensemble empirical mode decomposition (EEMD) [7,8], local mean decomposition (LMD) [9,10], and complete ensemble empirical mode decomposition with adaptive noise (CEEMDAN) [11,12]. Despite these advancements, all these methods still rely on recursive approaches for signal decomposition, which do not fundamentally overcome the challenge of mode aliasing. A variational mode decomposition (VMD) structure that does not require the use of recursive sieving has been proposed [13]. This technique sets the penalty factor $a_{0}$ and the modal breakdown number K. The denoising of the signal is achieved by addressing a variational problem. Yang et al. [14] utilized VMD to construct a new feature set and identified the bearing states by support vector machine (SVM) to obtain superior diagnostic results.

Another significant theoretical approach utilized in fault diagnosis is blind deconvolution (BD). The earliest BD algorithm is minimum entropy deconvolution (MED) [15]. Shao et al. [16] enhanced MED by combining kernel-based local outlier factor (KLOF) with phase editing (PE), significantly improving fault feature extraction in noisy environments. However, the MED method is influenced by individual pulses and does not account for the periodicity of fault signals during signal denoising. In response to these MED limitations, maximum correlation kurtosis deconvolution (MCKD) has been proposed to separate the cyclical shock components amid the severe background noise by finding an inverse filter that reduces signal noise and highlights the fault characteristics [17]. However, the MCKD method is limited by the need to set more parameters and its ability to extract only a finite number of local pulses, which restricts its scope of application [18].

The multipoint optimal minimum entropy deconvolution adjusted (MOMEDA) algorithm overcomes the drawbacks of MED and MCKD, providing an optimal filter without the need for iteration [19]. Despite enhancements to the algorithm, it is still limited to extracting only one periodic shock at a time. Furthermore, in instances of substantial noise interference, the periodic pulse isolated by the algorithm could potentially be a false component. In a more recent development, the maximum second-order cyclostationarity blind deconvolution (CYCBD) [20] algorithm was proposed, which seeks the optimal inverse filter utilizing the maximum second-order cyclic smoothness metrics. The algorithm’s capacity to pick up fault features is markedly more effective compared to earlier iterations of BD algorithms.

Although traditional methods are effective in troubleshooting tasks, they depend on experts’ knowledge and experiences to a large extent. Subsequently, deep learning (DL) [21] theory was proposed, which overcomes feature-extraction dependence on a priori knowledge. With the rapid development of artificial intelligence, researchers have greatly improved diagnostic performance by leveraging DL techniques. These include autoencoders (AE), convolutional neural networks (CNN), deep belief networks (DBN), and recurrent neural networks (RNN) [22]. Of these, the CNN—a classical DL approach—has been particularly instrumental in fault diagnosis. Zhang et al. [23] obtained good robustness under conditions such as small samples and load variations by converting the signal into the frequency domain and encoding the result as a 2D image combined with CNN to extract multiscale features. Chen et al. [24] integrated an approximation filter in the CNN’s initial layer to realize modulation and demodulation of useful components in different frequency bands and achieved superior performance in noisy environments.

CNNs are prone to a decline in performance recognition due to factors such as overfitting, gradient explosion, and class imbalance. Long short-term memory (LSTM) [25] networks are extensively applied in fault diagnosis owing to their proficiency in encoding temporal information and processing long-term sequential data. The LSTM and EEMD combinations utilize LSTM to extend the data to reduce the endpoint divergence degree. The processed signals are decomposed by EEMD, followed by a genetic algorithm to find the optimal weights to achieve high-accuracy diagnosis [26]. Wang et al. [27] implemented fault category classification by combining CEEMDAN and LSTM to decompose the signal and extract component signal features. However, although these two methods implement measures to reduce modal aliasing, it is still difficult to completely eliminate the modal aliasing phenomenon.

Existing methods struggle to extract weak fault data characteristics in a strong noise background. Additionally, preprocessing can only extract limited impulses. Furthermore, the classification of the extracted features depends on prior knowledge. To solve these issues, this paper proposes a method that integrates the denoising capability of CYCBD with that the feature extraction of bidirectional long short-term memory (BiLSTM) networks. The CYCBD method utilizes fault signal periodicity to extract fault characteristic information from the source signal, which contains strong background noise. To obtain suitable CYCBD parameters, the golden jackal optimization (GJO) algorithm is introduced to adaptively find the CYCBD parameters. The resulting denoised signals after the CYCBD processing are then used to train the BiLSTM network. The method was validated with the CWRU dataset. It compares the preprocessing part with three methods: VMD, MED, and MCKD, and compares the network part with multilayer perceptrons (MLP) and CNN. Lastly, the XJTU-SY bearing dataset was used for assessing the generalizability of the method. This paper’s principal contributions are threefold and may be summarized as follows:

1. The study object is the defective vibration signals of rolling bearings, and a GJO-CYCBD–BiLSTM fault detection method is suggested. The method uses CYCBD’s potent noise reduction capability to extract the features of faulty pulses, which are then classified by BiLSTM to achieve high-precision diagnosis.
2. The method optimizes the parameters of CYCBD by GJO, which fully utilizes the denoising benefits of GJO-CYCBD for noisy signals. Additionally, being a BD method, it highlights the signal’s pulse impact features while thoroughly considering the periodic properties of fault pulses, so it is advantageous to the BiLSTM feature acquisition process that follows.
3. In actual industrial production, the vibration characteristics are influenced by various background noises and bearing operating conditions. This presents an enormous challenge to fault diagnosis methods’ noise immunity and generalization. GJO-CYCBD–BiLSTM provides a feasible solution to these issues.

The rest of this paper is organized as follows. Section 2 describes CYCBD, GJO, and BiLSTM. In Section 3, we unfold the CYCBD–BiLSTM optimization model. Section 4 examines the algorithm’s proficiency in fault feature extraction. Section 5 confirms the efficacy of the suggested approach through experimental validation. Lastly, Section 6 summarizes the conclusions.

## 2. Related Method

### 2.1. Maximum Second-Order Cyclostationarity Blind Deconvolution

The CYCBD algorithm represents a novel method grounded in generalized Rayleigh entropy and iterative eigenvalue decomposition, enabling the efficient extraction of periodic pulse features [20]. Functioning as a BD method, CYCBD aims to find the fault signatures from the original signal x. The deconvolution process is described below:

(1) $$s=x⊗h=\left(s_{0}⊗g\right)⊗h\approx s_{0}$$

where s is the input original signal, x is the hybrid signal, h is the inverse filter, ⊗ denotes the convolution operation, $s_{0}$ is the estimated source signal, and g is the unknown impulse response function.

The matrix form of Equation (1) is put as follows:

(2) s=Xh

Which is

(3) $$\left[\begin{array}{c} s\left[L-1\right]\\ \vdots \\ s\left[N-1\right] \end{array}\right]=\left[\begin{array}{ccc} x\left[L-1\right] & \cdots & x\left[0\right]\\ \vdots & \ddots & \vdots \\ x\left[N-1\right] & \cdots & x\left[N-L-2\right] \end{array}\right]\left[\begin{array}{c} h\left[0\right]\\ \vdots \\ h\left[L-1\right] \end{array}\right]$$

where N is the length of the signal s and L is the length of the filter h.

And the second-order cyclic smoothness index can be defined as:

(4) $$ICS_{2}=\frac{h^{H}X^{H}WXh}{h^{H}X^{H}Xh}=\frac{h^{H}R_{XWX}h}{h^{H}R_{XX}h}$$

where H denotes matrix conjugate transpose; $R_{XWX}$ and $R_{XX}$ represent the weighted correlation matrix and the correlation matrix, respectively; W is a weighted matrix and has:

(5) $$W=diag\left(\frac{P{\left|s\right|}^{2}}{s^{H}s}\left(N-L+1\right)\right)=\left[\begin{array}{ccc} \ddots & & 0\\ & P{\left|s\right|}^{2} & \\ 0 & & \ddots \end{array}\right]\frac{\left(N-L+1\right)}{\sum_{l=L-1}^{N-1}{\left|s\right|}^{2}}$$

(6) $$P\left[{\left|s\right|}^{2}\right]=\frac{1}{N-L+1}\sum_{k}e_{k}\left(e_{k}^{H}{\left|s\right|}^{2}\right)=\frac{EE^{H}{\left|s\right|}^{2}}{N-L+1}$$

(7) $$E=\left[\begin{array}{ccc} e^{-j2\pi \frac{1}{T_{s}}(L-1)} & \cdots & e^{-j2\pi \frac{K}{T_{s}}(L-1)}\\ \vdots & \ddots & \vdots \\ e^{-j2\pi \frac{1}{T_{s}}(N-1)} & \cdots & e^{-j2\pi \frac{K}{T_{s}}(N-1)} \end{array}\right]$$

where $P\left[{\left|s\right|}^{2}\right]$ denotes the feature matrix including the failure cycle frequency. $T_{s}$ is the failure cycle frequency, which is associated with mechanical equipment failures such as bearings. *k* is the sample size. Thus, the following is the set of discrete-time signal cycle frequencies:

(8) $$\alpha =\frac{k}{T_{s}}$$

The maximum ${ICS}_{2}$ value is found by solving the generalized eigenvalues, and the maximum eigenvalue λ corresponds to the maximum ${ICS}_{2}$ value, which can be calculated as follows:

(9) $$R_{XWX}h=R_{XX}h\lambda$$

As the weighting matrix W is initialized by estimating the initial filter h, the maximum value of ${ICS}_{2}$ should be obtained iteratively through the process outlined below:

(1) Initialize the filter h;
(2) Compute the weighted matrix W using the input signal X and the filter h.
(3) Solve for the maximum eigenvalue λ and the corresponding filter h via Equation (9);
(4) Return to step (2) and recalculate through the obtained h until convergence.

The iteration stops when the number of iterations k reaches the maximum iteration value K=100 or when $\Delta {ICS}_{2}<\varepsilon$.

### 2.2. Golden Jackal Optimization

The GJO is a new intelligent optimization algorithm that is inspired by the golden jackal’s collaborative prey hunting process and was thus proposed. The algorithm well simulates the golden jackal’s grasp of opportunities during hunting to achieve the effect of being able to adapt to hunting in different environments, with fast optimization search and good convergence performance [28].

The initialization of the GJO on the search space is mathematically described as:

(10) $$Y_{0}=Y_{\min}+rand\times \left(Y_{\max}-Y_{\min}\right)$$

where $Y_{0}$ is the initial position of the golden jackal population; rand is a random number in [0, 1]; and $Y_{max}$ and $Y_{min}$ are the maximum and minimum positions in the golden jackal population, respectively, which correspond to the upper and lower boundaries of the question to be solved.

In the GJO, the number of prey was assumed to be n and the variable dimension of the issue being solved is d. $Y_{i,j}$ denotes the position of the i-th prey in the j-th dimension of the space, i=1,2,…,n,j=1,2,…,d. Then, the matrix of prey locations is:

(11) $$Prey=\left[\begin{array}{ccccc} Y_{1,1} & \cdots & Y_{1,j} & \cdots & Y_{1,d}\\ \vdots & \ddots & \vdots & & \vdots \\ Y_{i,1} & \cdots & Y_{i,j} & \cdots & Y_{1,d}\\ \vdots & & \vdots & \ddots & \vdots \\ Y_{n,1} & \cdots & Y_{n,j} & \cdots & Y_{n,d} \end{array}\right]$$

The fitness of each prey during the iterative process is calculated based on the fitness function, and the prey fitness value matrix is:

(12) $$F=\left[\begin{array}{c} f\left(Y_{1,1};Y_{1,2};\cdots ;Y_{1,d}\right)\\ f\left(Y_{2,1};Y_{2,2};\cdots ;Y_{2,d}\right)\\ \vdots \\ f\left(Y_{n,1};Y_{n,2};\cdots ;Y_{n,d}\right) \end{array}\right]$$

where f(·) is the fitness function, the optimal fitness is considered to be the male jackal and the sub-optimal is the female jackal, the optimal fitness and the sub-optimal fitness form the golden jackal pair. The corresponding prey’s location can be obtained through this pair of golden jackals.

The golden jackals’ foraging instincts enable them to adeptly detect and pursue their quarry. However, capturing prey is not always straightforward, compelling the jackals to exhibit patience and entertain alternative hunting targets. During the pursuit, the leading role is adopted by the male jackal, thus influencing the subsequent movements of the female jackals. This behavior can be expressed as:

(13) $$Y_{1}\left(t\right)=Y_{M}\left(t\right)-E\cdot \left|Y_{M}\left(t\right)-l\cdot prey\left(t\right)\right|$$

(14) $$Y_{2}\left(t\right)=Y_{FM}\left(t\right)-E\cdot \left|Y_{FM}\left(t\right)-l\cdot prey\left(t\right)\right|$$

In the t-th iteration, $Y_{M}(t)$ and $Y_{FM}(t)$ represent male and female jackal locations, respectively. $Y_{1}(t)$ and $Y_{2}(t)$ represent the adjusted male and female jackal locations, respectively, after interaction with prey. E represents the prey’s escape energy. The golden jackal population engages in the search phase when the magnitude of $\left|E\right|$ is greater than 1; conversely, when the magnitude of $\left|E\right|$ is less than or equal to 1, the population transitions into the attack phase. The escape energy E is determined by the following calculation:

(15) $$E=E_{1}*E_{0}$$

where $E_{1}$ represents the decreasing degree of prey escape energy and $E_{0}$ represents the initial energy level at which the prey escapes. It could be calculated using the following equations, respectively:

(16) $$E_{0}=2*r-1$$

(17) $$E_{1}=c_{1}*\left(1-\frac{t}{T}\right)$$

where r is the random value between [0, 1], $c_{1}$ is a constant with a value 1.5, t and T are the current iteration number and maximum iteration number, respectively, and then update the golden jackal’s position using the following equation:

(18) $$Y\left(t+1\right)=\frac{Y_{1}\left(t\right)+Y_{2}\left(t\right)}{2}$$

l denotes a random value based on the Lévy distribution with the following mathematical formula:

(19) $$l=0.05*LF\left(y\right)$$

LF is a random value in the Lévy distribution, calculated as:

(20) $$LF\left(y\right)=0.01\times \frac{\mu \times \sigma }{\left|\nu^{\left(\frac{1}{\beta }\right)}\right|};\sigma ={\left[\frac{\Gamma \left(1+\beta \right)\times \sin\left(\frac{\pi \beta }{2}\right)}{\Gamma \left(\frac{1+\beta }{2}\right)\times \beta \times \left(2^{\frac{\beta -1}{2}}\right)}\right]}^{\frac{1}{\beta }}$$

where μ and υ are random numbers between (0, 1), β is a constant with a value of 1.5, and Γ(·) denotes the gamma function.

In the process of prey capture by golden jackal pairs, the prey’s escape energy gradually decreases. Subsequently, male and female jackals surround and approach the prey. This scenario is mathematically modeled below:

(21) $$Y_{1}\left(t\right)=Y_{M}\left(t\right)-E\cdot \left|l\cdot Y_{M}\left(t\right)-prey\left(t\right)\right|$$

(22) $$Y_{2}\left(t\right)=Y_{FM}\left(t\right)-E\cdot \left|l\cdot Y_{FM}\left(t\right)-prey\left(t\right)\right|$$

In every iteration of the GJO, the male and female jackals jointly forecast their target’s probable position and then adjust the distance between pairs of jackals to follow the prey according to set criteria.

**Figure 1:**
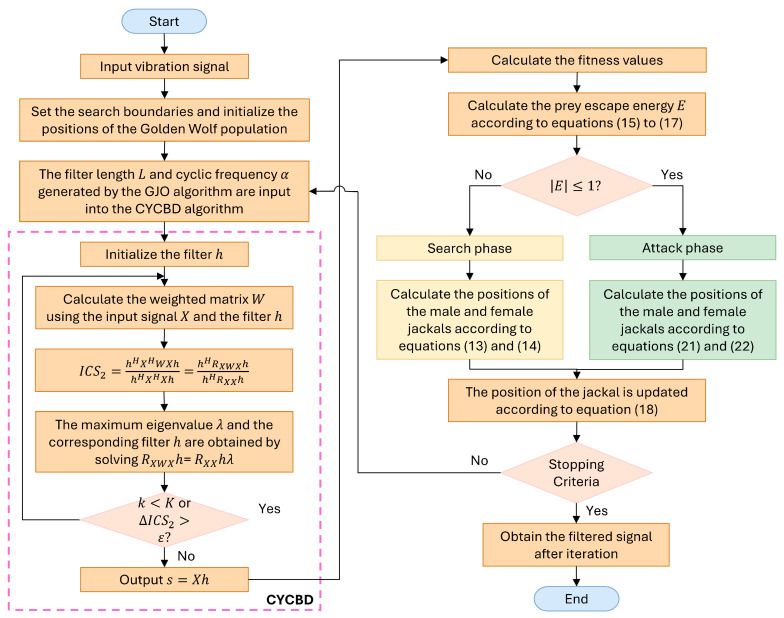
GJO-CYCBD algorithm flowchart.

The fault feature separation process based on the GJO-CYCBD algorithm is shown in Figure 1, and the specific steps are as follows:

Step 1: Input the vibration signal, set the search boundaries, and initialize the positions of the golden jackal pack.

Step 2: Input the filter length L and cycling frequency α into the CYCBD algorithm, perform the deconvolution operation described in Section 2.1, and extract the corresponding deconvolution signal.

Step 3: Calculate the fitness value and the prey’s escape energy.

Step 4: Based on the escape energy, enter the corresponding search or attack phase, while calculating the positions of the male and female jackals. Update the jackal positions.

Step 5: Repeat steps (2) to (4) until the maximum number of iterations is reached.

Step 6: Substitute the optimized parameters *L* and α into the CYCBD algorithm to obtain the filtered signal after iteration.

### 2.3. Deep Learning Classification Models

**Figure 2:**
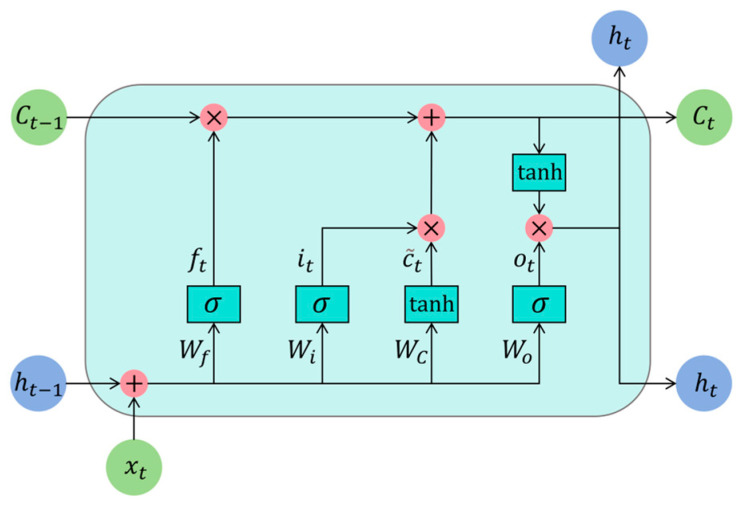
LSTM architecture.

LSTM is an improved RNN that solves the issue of gradient vanishing and explosion during long time-series training by introducing a gate mechanism to remember or delete information. Each LSTM module contains three “gating” structures, namely the input gate, the output gate, and the forget gate. Through the gate structure, it adds or removes information from each memory block and learns the long-distance dependency information [25]. The LSTM network’s memory block architecture is illustrated in Figure 2.

The LSTM is calculated as follows:

(23) $$\left\{\begin{array}{c} f_{t}=\sigma \left(W_{f}\cdot \left[h_{t-1},x_{t}\right]+\beta_{f}\right)\\ i_{t}=\sigma \left(W_{i}\cdot \left[h_{t-1},x_{t}\right]+\beta_{i}\right)\\ {\tilde{C}}_{t}=\tanh\left(W_{C}\cdot \left[h_{t-1},x_{t}\right]+\beta_{C}\right)\\ C_{t}=f_{t}*C_{t-1}+i_{t}*{\tilde{C}}_{t}\\ \begin{array}{c} o_{t}=\sigma \left(W_{o}\cdot \left[h_{t-1},x_{t}\right]+\beta_{o}\right)\\ h_{t}=o_{t}*\tanh\left(C_{t}\right) \end{array} \end{array}\right.$$

where $f_{t}$ represents the forgetting gate’s output, and $h_{t-1}$ and $x_{t}$ correspond to the output and input at times t−1 and t, respectively. $W_{f}$, $W_{i}$, $W_{C}$, and $W_{o}$ are weight matrices, while $\beta_{f}$, $\beta_{i}$, $\beta_{C}$, and $\beta_{o}$ are bias matrices. $i_{t}$ stands for the input gate’s output, ${\tilde{C}}_{t}$ is the candidate state vector, and $C_{t}$ represents the updated memory cell. The states of the memory cell at times t and t−1 are expressed as $C_{t}$ and $C_{t-1}$, respectively. The symbols σ and tanh indicate the nonlinear activation functions employed.

**Figure 3:**
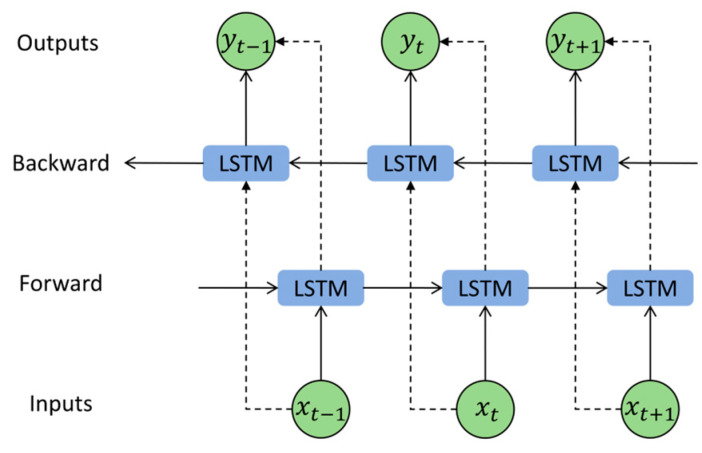
BiLSTM network structure.

The traditional LSTM network is trained in a forward-only manner. This unidirectional training method has a relatively low feature extraction capability. The BiLSTM consists of both forward and backward channels, as shown in Figure 3. This network structure enhances the ability to extract data features by considering both past and future states, thus uncovering relationships between data.

## 3. An Optimized CYCBD–BiLSTM Model for Rolling Bearing Fault Diagnosis

Considering that bearings are influenced by adverse conditions such as mechanical equipment noise during operation, the actual bearing data collected from industrial environments often contains significant multi-frequency background noise. Therefore, preprocessing the collected signals to minimize the impact of interference components can improve diagnostic accuracy in fault diagnosis. When a bearing fails, a periodic pulse component appears in the vibration signal. This characteristic allows for the separation of fault feature components from noise components within the signal.

As introduced in the previous sections, this paper uses the GJO optimization algorithm to adaptively determine the filter length L and cyclic frequency α in CYCBD. Therefore, an appropriate fitness function is necessary to evaluate the optimal solution. This paper uses the crest factor of the envelope spectrum (Ec) introduced in the literature [29] as the fitness function. Ec considers the impact and vibration signal amplitude. The Ec indicator can be defined as:

(24) $$\mathrm{Ec}=\frac{\max\left(X\left(j\right)\right)}{\sqrt{\frac{\sum_{j}X{\left(j\right)}^{2}}{M}}}$$

X(j)(j=1,2,...,M) denotes the amplitude of the signal envelope spectrum frequency range within [$f_{r^{\prime }}$, $n*f_{i^{\prime }}$], where $f_{r^{\prime }}$ is slightly greater than the bearing’s shaft rotation frequency, and $f_{i^{\prime }}$ is the bearing’s maximum fault frequency. Here, n is a constant, set to 8 in this study. The index Ec is used to measure whether the parameters found by the optimization algorithm are applicable; the larger the Ec, the more distinct the periodic impact characteristics. After filtering, the signal has a higher signal-to-noise ratio (SNR) and greater pulse energy than the raw one, making the fault features more prominent. Since CYCBD can highlight the characteristics of pulse impacts through second-order cyclostationary indicators, using Ec as the CYCBD optimization fitness function can effectively evaluate if the parameter selection is suitable.

From current research, intelligent optimization algorithms are extensively applied for optimizing different parameters in the fault diagnosis process. The GJO, in particular, has advantages such as simple principles, few parameter settings, and fast computation speed. It performs excellently in benchmark function testing and fault parameter optimization, enabling researchers to find the optimal combination of parameters more quickly without relying on prior knowledge.

In industrial processes, the bearing fault data collected by sensors often contain significant noise, which can affect diagnostic accuracy to varying degrees. To confirm that the proposed method is robust, this experiment adds Gaussian white noise with varying SNR to the input signal. The definition of SNR is given in Equation (25).

(25) $${\mathrm{SNR}}_{dB}=10\log_{10}\left(\frac{p_{signal}}{p_{noise}}\right)$$

where $p_{signal}$ and $p_{noise}$ represent the raw signal power and noise signal power, respectively. When the SNR is negative, the signal’s noisy component has a higher power than its useful component; conversely, when the SNR is positive, the power of the useful part is greater. When the SNR is zero, the useful part of the signal has the same power as the useless part. The lower the SNR, the more noise the signal contains.

**Figure 4:**
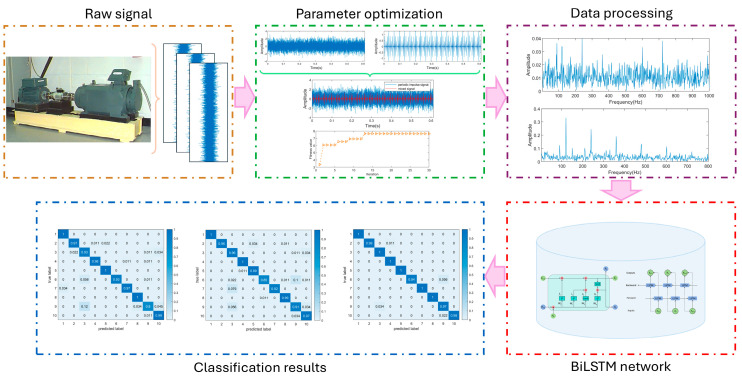
The fault diagnosis process of the proposed method.

**Figure 5:**
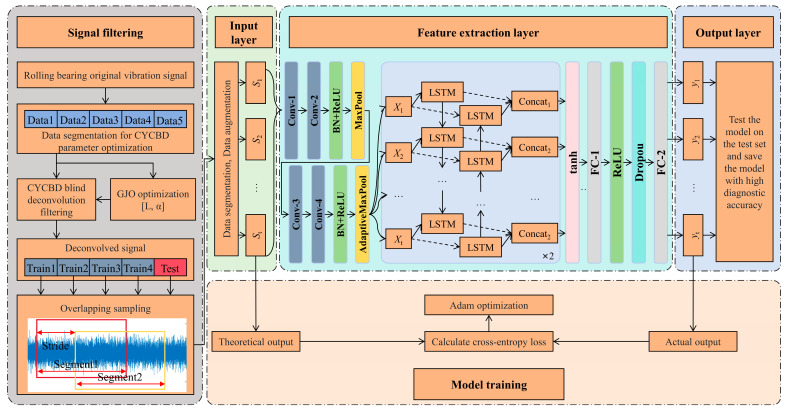
Flowchart of rolling bearing fault diagnosis based on a CYCBD–BiLSTM approach.

Due to the strong temporal nature of the collected vibration signals, and given that BiLSTM is a neural network well suited for processing sequential data, it is employed for fault classification of the vibration data processed by GJO-CYCBD. The data processing flow is shown in Figure 4. Initially, the vibration signal is input, and the filter length L and cycle frequency α are optimized. The optimized parameters are then fed into the CYCBD algorithm for data processing. Next, the processed data are used to train and test the BiLSTM network, ultimately producing classification results for fault diagnosis. The overall framework, as shown in Figure 5, consists of five parts: signal filtering, input layer, hidden layers, model training, and output layer. The optimal parameter combination of CYCBD is obtained by parameter optimization and denoises the source signal with BD filtering. After that, the signal is converted to the frequency domain and input into the model. The input layer performs overlapping sampling on the data. The feature extraction layer contains two layers of BiLSTM and the network parameters are shown in Table 1. The operating condition with the highest predicted probability is selected as the diagnostic result. Due to its strong adaptability and fast convergence speed, the Adam optimizer has become one of the most commonly used optimizers in neural network models. Therefore, the Adam optimizer is employed for model training in this paper. The overall architecture of the model is shown in Figure 5.

## 4. Algorithm Effectiveness Analysis

Periodic shock components are included within the collected vibration signal when bearing damage occurs. This section verifies the validity of CYCBD on fault feature extraction using simulated signals. The simulated signals are a set of discrete signals that simulate an inner ring fault in a wind turbine bearing [30]. The simulated signals include fault-induced impact signals and high-energy Gaussian white noise, simulating the weak fault signals and strong background noise produced during bearing operation. The simulated signals are as follows:

(26) $$\left\{\begin{array}{c} x\left(t\right)=y\left(t\right)+n\left(t\right)=\sum_{k}A_{k}h\left(t-kT_{1}-\tau_{k}\right)+n\left(t\right)\\ A_{k}=A_{0}\sin\left(2\pi f_{r}t\right)+1\\ h\left(t\right)=\exp\left(-Ct\right)\sin\left(2\pi f_{n}t\right) \end{array}\right.$$

**Figure 6:**
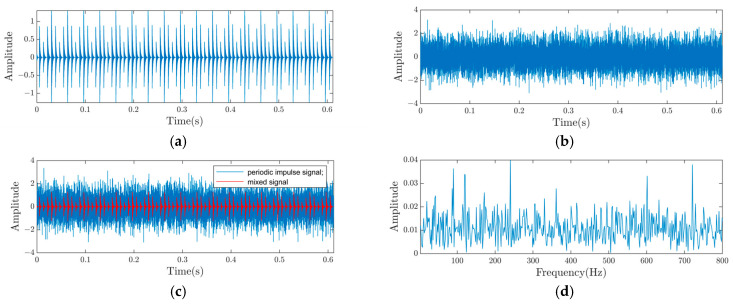
Simulated signal waveform and spectrum. (**a**) The time-domain waveform of the fault signal; (**b**) the time-domain waveform of the noise signal; (**c**) the time-domain waveform of a mixed-signal system; (**d**) the envelope spectrum of the mixed signal.

The equation simulates the inner race fault of a rolling bearing. The parameters are set as follows: $A_{0}=0.8$, the sampling frequency $f_{s}$ is 25.6 kHz, and the rotational frequency $f_{r}$ is 30 Hz. $T_{1}$ is 1/120 s, setting the inner race fault frequency $f_{i}$ to 120 Hz. The natural frequency $f_{n}$ is 4000 Hz. $\tau_{\kappa }$ denotes the fluctuation of the κ-th impact relative to the period T, with the random sliding following a normal distribution, mean of 0, and a standard deviation of 0.5% of the rotational frequency. The decay coefficient C is 800. A signal with an SNR of −12 dB is obtained by adding Gaussian white noise n(t) to the signal. The signal is sampled with 15,360 points. The time domain plots of the shock signal and the additional noise signal are displayed in Figure 6a and Figure 6b, respectively. Figure 6c,d show the spectrum and envelope spectrum of the noise-added signal, respectively. The periodic shocks are easily seen in Figure 6a,c,d demonstrate that the signal after adding noise cannot be directly analyzed through traditional methods.

**Figure 7:**
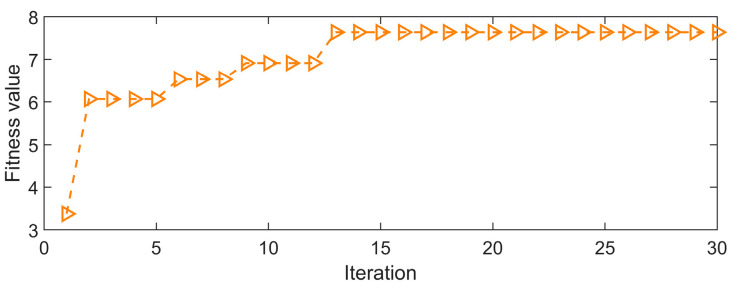
The curve of Ec with population iteration.

The simulated signals are processed by the GJO-CYCBD method. Firstly, the parameters [L, α] in CYCBD are optimized. Considering that an increase in filter length extends the running time of CYCBD, the filter length is searched within the range [50, 300], and the cyclic frequency within [90, 180]. The envelope spectrum peak factor’s frequency range for the envelope spectrum is set between [50, 800]. The population size is 20, and the maximum iterations number is 30. Figure 7 illustrates the curve of the envelope spectrum peak factor Ec with population iterations. The horizontal ordinate is the iteration count and the vertical ordinate is the fitness Ec value.

**Figure 8:**
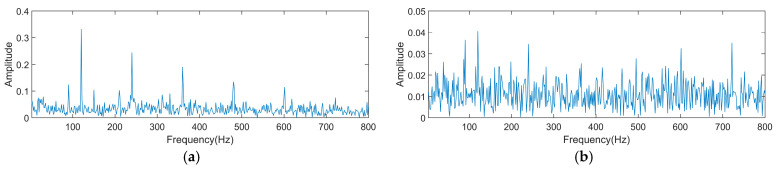
Envelope spectrum post-CYCBD processing. (**a**) The envelope spectrum after processing with optimal CYCBD parameters; (**b**) the envelope spectrum of the signal after modifying optimization parameters.

From Figure 7, it is observed that the GJO converges at the 13th iteration. At this point, the optimal filter length and cyclic frequency combination obtained from the optimization search is [64, 120], and the Ec value found is 7.6392. Therefore, the CYCBD filter length is placed at 64 and the cyclic frequency is placed at 120. Figure 8a depicts the signal’s envelope spectrum following CYCBD processing.

After deconvolution, the fault eigenfrequencies of the signal and their integer multiples are apparent in the envelope spectrogram. The feature frequency is accurately extracted, demonstrating that adaptive CYCBD is feasible for extracting fault characteristic frequencies. To compare with the situation where parameter settings are inappropriate, the filter length and cyclic frequency were altered by 10%, setting the parameter combination to [70, 108]. Figure 8b displays the processed signal’s envelope spectrum. From the image, it is evident that although the fault frequency can be seen, there is significant interference around it. The fault eigenfrequencies and their integer multiples are not prominent enough for accurate identification. This indicates the necessity of optimizing the CYCBD.

## 5. Experimental Results and Analysis

**Figure 9:**
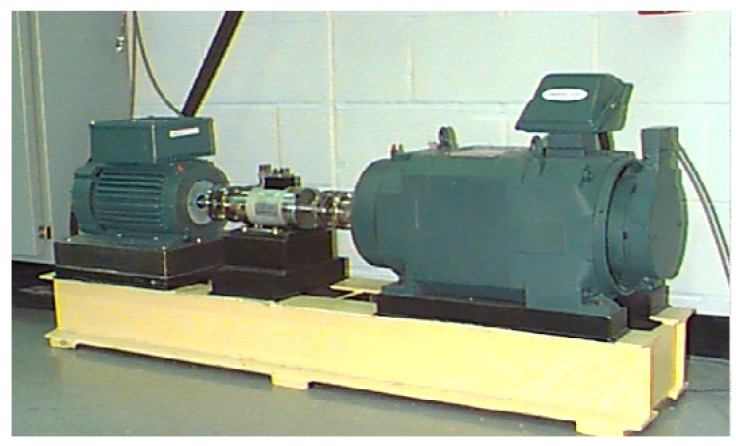
CWRU testing rig.

The experimental platform for algorithm comparison was MATLAB R2021a, the neural network part was written based on the PyTorch 1.13 framework, and the case study was run on a computer equipped with an Intel i9-12900K CPU. To validate the effectiveness of the experimental methodology, experiments are conducted with the dataset from the Bearing Data Center of Case Western Reserve University (CWRU) [31]. The data collection apparatus, depicted in Figure 9, includes a two-horsepower motor, torque sensor, and dynamometer. Bearing failures within the dataset were induced via electric spark machining. The research employed data from drive ends with faults sized at 0.007, 0.014, and 0.021 inches, under operational conditions set to a motor speed of 1797 rpm, with a zero load. The data were sampled at 12 kHz. The experiments used different operating conditions: three types of inner race (IR), outer race (OR), and ball faults, along with normal condition (NOR) of the rolling bearing.

In this study, 117,760 data points were selected from each of the ten experimental working conditions. 80% of the data are used as a training set and 20% as a test set. The time complexity for calculating the weighted correlation matrix in the CYCBD filtering process is approximately given by $(M+1)NL^{2}$, with M representing iteration number, N the signal length, and L the filter length [32]. Excessively lengthy datasets lead to prohibitive computational time complexity. Conversely, a smaller dataset size negatively impacts the robustness of BD by becoming overly influenced by local data during filter length and loop frequency optimization. To address this, the data are partitioned into five equal-sized segments, resulting in a data length of 23,552 points per segment for CYCBD processing. Additionally, to ensure that there are sufficient samples for training and testing, the data undergo overlap sampling before being inputted into the BiLSTM network. The overlapped sample has a length of 1024 with a stride of 256. The associated classification information is detailed in Table 2.

### 5.1. Learning Rate Selection

**Figure 10:**
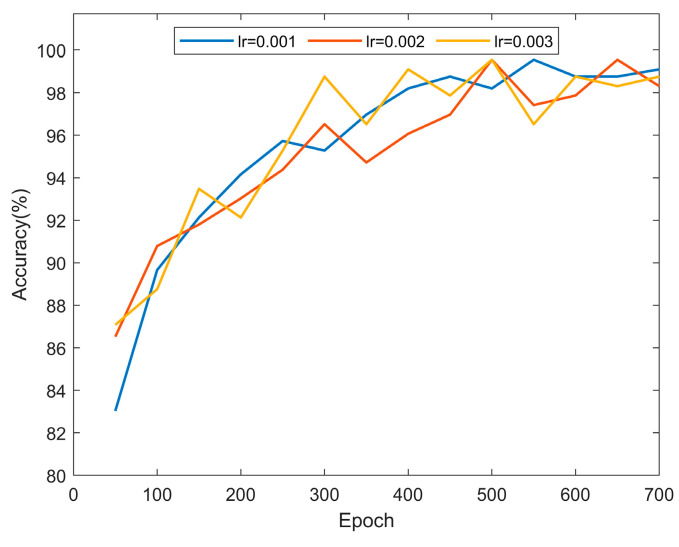
Comparison of diagnostic accuracy of BiLSTM at different learning rates.

During the training of the BiLSTM network, the learning rate significantly impacts the network’s performance. For comparison, learning rates of 0.001, 0.002, and 0.003 were selected, with the maximum training iterations set at 700, recording results in every 50 iterations. As shown in Figure 10, all three learning rates exhibit good accuracy. However, after 400 iterations, it is evident from the change in the curve’s slope that with initial learning rates set at 0.002 and 0.003, there were relatively significant fluctuations in diagnostic accuracy during the iterations. Therefore, a learning rate of 0.001 was chosen for all experiments in this study.

### 5.2. Data Preprocessing Methods

In this section, various degrees of noise will be added to the signal and various data processing methods will be used to preprocess the noisy signal. The processed signals will then be used as inputs for training and testing the BiLSTM model. Table 3 lists the data processing methods and optimization parameter settings.

**Figure 11:**
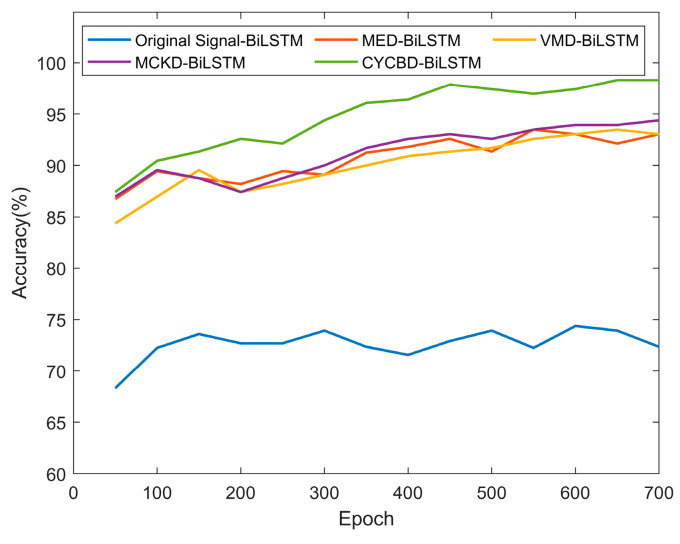
Comparison of fault diagnosis accuracy after different preprocessing.

Adding noise with −6 dB SNR in the dataset. Using the aforementioned optimization settings, the parameters for the MED, MCKD, VMD, and CYCBD methods were optimized, and a set of unprocessed data was included for comparison. These five datasets were then input to BiLSTM networks to train and test the model. By monitoring the changes in diagnostic accuracy as the number of iterations increased, the impact of different data preprocessing methods on diagnostic accuracy within the same model was compared. The outcomes are depicted in Figure 11.

Background noise in vibration signal analysis significantly compromises diagnostic accuracy when such signals are input directly into a model. Figure 11 demonstrates that when −6dB noise is added to a signal, diagnostic accuracy plunges to 72.36% without preprocessing. In contrast, implementing any preprocessing techniques listed in Table 3 results in an improvement of over 20% in diagnostic accuracy, highlighting the crucial role of signal preprocessing. The MED, an early BD algorithm, shows comparable performance to the VMD method, with both achieving diagnostic accuracies above 93%. This equivalence accentuates the effectiveness of BD techniques in fault diagnosis. The MCKD method enhances diagnostic accuracy by an additional 1.0% over MED and VMD at this noise level. However, it is found that the effect of MCKD is relatively unstable in many experiments, which may be due to the high optimization difficulty caused by the large number of parameters it needs to optimize. Utilizing the novel approach prescribed in this study, the diagnostic accuracy after 100 iterations markedly surpasses that of alternative methods, reaching upwards of 98% for the optimized model.

**Figure 12:**
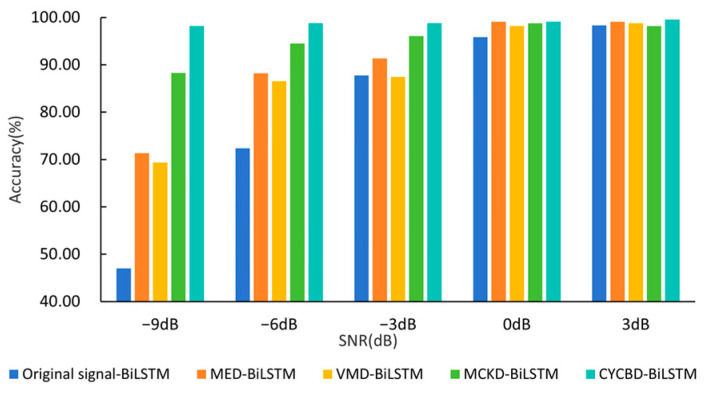
Experimental results under different noise levels.

To evaluate the effectiveness of preprocessing methods under various noise levels, this experiment introduces noise with different SNRs into the signal. The signals obtained after different preprocessing were then fed to the BiLSTM network with the diagnostic outcomes presented in Figure 12. As shown in the graph, the noise immunity of different signal processing methods varies. Without any signal processing, model diagnostic accuracy starts to decrease rapidly after adding Gaussian white noise with an SNR below 0 dB. At −9 dB, the diagnostic accuracy drops to only 46.97%, which is insufficient for meeting the accuracy requirements of practical engineering fault diagnosis. MED and VMD show comparable performance in fault diagnosis. Although they are advantageous compared to no processing, their diagnostic performance significantly declines after 0 dB, decreasing to 88.2% at an SNR below −6 dB. The signal preprocessed by MCKD demonstrates a notable advantage when the SNR is below −3 dB, maintaining an accuracy of 88.31% at −9 dB. The proposed method gives superior results at various SNRs, with a diagnostic accuracy of 98.20% even at an SNR of −9 dB, which is approximately 9.89% improvement in accuracy, surpassing other preprocessing methods.

### 5.3. Comparison of Diagnostic Accuracy of Neural Networks

To further verify the CYCBD–BiLSTM framework, CYCBD is combined with MLP and CNN networks, respectively, and the three models are compared. The diagnostic accuracy is tested at SNR of −9, −6, −3, 0, 3, 6, and 9. Due to fluctuations during the training of neural networks, each experiment is repeated five times. The models were assessed through the comparison of the best accuracy, the last epoch’s best accuracy, and the last epoch’s average accuracy under different SNRs.

**Figure 13:**
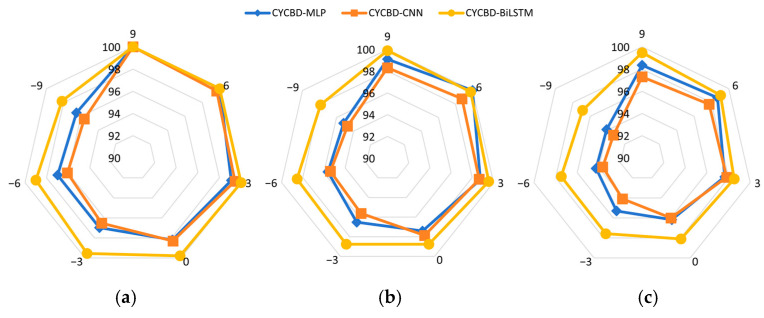
Performance of different models in diagnosis. (**a**) Best precision of the model; (**b**) best precision of the last epoch; (**c**) average precision of the last epoch.

**Figure 14:**
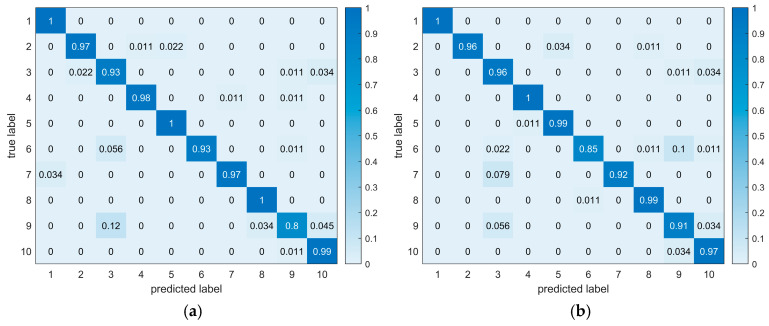

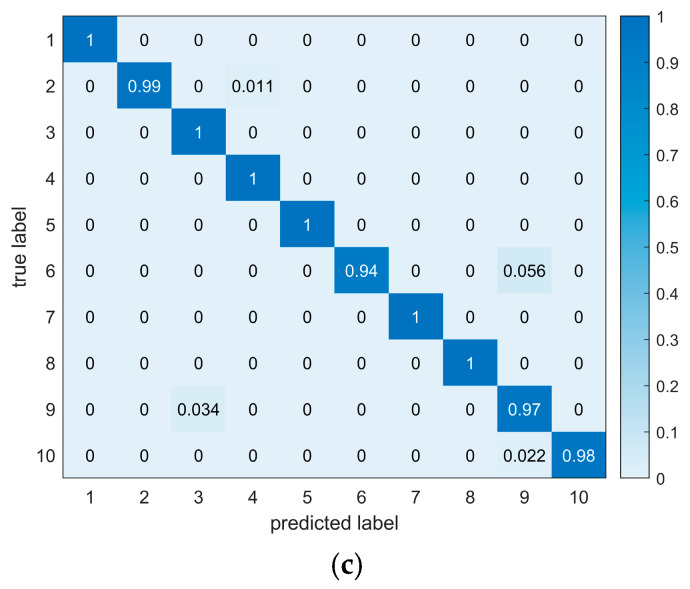
Confusion matrix of classification accuracy of the network model for CWRU data at SNR −6 dB. (**a**) Classification results of the last epoch of CYCBD-MLP; (**b**) classification results of the last epoch of CYCBD-CNN; (**c**) classification results of the last epoch of CYCBD–BiLSTM.

The performance of the three models is shown in Figure 13. It is observed that when the SNR is high, indicating low noise in the signal, all three models perform well. When the SNR is below 0 dB, the proposed method shows a remarkable advantage in diagnostic precision across three metrics compared with CYCBD-MLP and CYCBD-CNN. Notably, in the low SNR comparison of average accuracy in the last epoch, the other two models exhibited large fluctuations in accuracy during the experiments, leading to low-accuracy diagnoses that reduced the average accuracy, with CNN being particularly affected. To further compare the models’ diagnostic performance of various types of faults, Figure 14 visualizes the classification situation for the last epoch with an SNR of −6 dB. The proposed method shows a stronger classification ability of fault modes, with fewer misdiagnoses compared to CYCBD-MLP and CYCBD-CNN. In the diagnostic experiments conducted with this dataset, the proposed method improved diagnostic performance by approximately 3% compared to the other two methods.

### 5.4. XJTU-SY Bearing Dataset Validation

To validate that the model is valid for various operating conditions, experiments are conducted using the XJTU-SY bearing dataset [33]. The dataset details are given in Table 4. Because of the sizable dataset, only the last five test cycles for each operating condition were used, with 29,696 data points per test cycle, an overlapping sample length of 1024, and an interval of 256. “Best Max” represents the model’s best accuracy, “Last Max” represents the best accuracy in the last epoch, and “Last Mean” represents the average accuracy in the last epoch. The performance of CYCBD–BiLSTM on the XJTU-SY bearing dataset under different SNRs is shown in Table 5.

The proposed method continues to achieve good results on the XJTU-SY bearing dataset. Even at an SNR of −9 dB, the model’s best accuracy remains above 98%, the best accuracy in the last epoch is close to 98%, and the average accuracy in the last epoch reaches 96.67%. This indicates that CYCBD–BiLSTM performs well and is highly robust under various conditions.

## 6. Conclusions

To address the low rolling bearing fault diagnosis precision under strong background noise, a BiLSTM model with GJO-optimized CYCBD parameters is proposed to diagnose different rolling bearings’ health states. This method starts from the collected raw signals and uses the GJO to adaptively find two parameters of the CYCBD: filter length and cyclic frequency. The optimized parameters are then incorporated into the CYCBD algorithm to denoise the signals and amplify the periodic shock constituents of the signals. Subsequently, the frequency domain signals are utilized as inputs to categorize the fault diagnosis by the BiLSTM model. By comparing the changes in the envelope spectra of the simulated signals before and after GJO-CYCBD denoising, the validity of CYCBD in extracting fault frequencies is demonstrated. As the learning rate significantly affects the model, the CWRU public dataset is utilized to compare the impact of different learning rates on the diagnosis, with 0.001 selected as the model’s learning rate. Comparisons are made between different preprocessing methods and model combinations for demonstrating the CYCBD–BiLSTM method’s efficacy. The generalizability of this method is validated through the XJTU-SY bearing dataset. Results show that CYCBD–BiLSTM has better noise immunity compared to other advanced fault diagnosis methods. Its advantages in accuracy and stability can provide new insights for rolling bearing fault diagnosis under strong background noise. Meanwhile, the framework is theoretically capable of diagnosing fault signals in metallic, hybrid, and ceramic bearings. However, since the method requires signal preprocessing, it may introduce additional errors during processing. Therefore, future research will focus on further implementing end-to-end modeling, while adopting more advanced network architectures to fully leverage the potential of neural networks for better fault feature extraction and fault type diagnosis.

## Figures and Tables

**Table 1:** Main network structure parameters.

Layer	Kernel/Stride/Padding	Output
Conv layer-1	3/1/1	64 × 16 × 512
Conv layer-2	3/1/1	64 × 16 × 512
MaxPool	2/2/0	64 × 16 × 256
Conv layer-3	3/1/1	64 × 32 × 256
Conv layer-4	3/1/1	64 × 32 × 256
AdaptiveMaxPool	/	64 × 32 × 25
BiLSTM layer-1	/	64 × 128 × 25
BiLSTM layer-2	/	64 × 128 × 25
FC layer-1	/	64 × 256
FC layer-2	/	64 × 10

**Table 2:** Details of CWRU experimental data.

Label	Damage Diameter /mm	Fault Type	Number of Samples	Training/ Validation
1	0	NOR	445	356/89
2	0.1778	IR	445	356/89
3	0.1778	OR	445	356/89
4	0.1778	Ball	445	356/89
5	0.3556	IR	445	356/89
6	0.3556	OR	445	356/89
7	0.3556	Ball	445	356/89
8	0.5334	IR	445	356/89
9	0.5334	OR	445	356/89
10	0.5334	Ball	445	356/89

**Table 3:** Optimized settings for data preprocessing methods.

Method	Optimization Parameter	Number of Dimensions	Lower Limit	Upper Limit	Maximum Iterations
MED	Filter length L	1	[100]	[1000]	20
VMD	$\mathrm{Penalty}\;\mathrm{factor}\;a_{0}$ , number of components K	2	[300 3]	[2500 10]	20
MCKD	Filter length L , deconvolution period T , number of shifts M	3	[100 60 1]	[1000 200 7]	20
CYCBD	Filter length L , cycle frequency α	2	[50 90]	[300 180]	20

**Table 4:** Details of XJTU-SY experimental data.

Label	Operating Condition	Fault Type	Number of Samples	Training/ Validation
1	35 Hz 12 kN	OR	565	452/113
2	35 Hz 12 kN	OR	565	452/113
3	35 Hz 12 kN	OR	565	452/113
4	35 Hz 12 kN	Cage	565	452/113
5	35 Hz 12 kN	IR + OR	565	452/113
6	37.5 Hz 11 kN	OR	565	452/113
7	37.5 Hz 11 kN	IR + Ball + Cage + OR	565	452/113
8	37.5 Hz 11 kN	IR	565	452/113
9	37.5 Hz 11 kN	IR	565	452/113
10	37.5 Hz 11 kN	OR	565	452/113

**Table 5:** Diagnostic performance of CYCBD–BiLSTM on the XJTU-SY bearing dataset.

SNR(dB)	−9	−6	−3	0	3	6	9
Best Max (%)	98.43	98.88	99.55	99.55	100.00	100.00	100.00
Last Max (%)	97.98	98.43	98.20	98.65	99.33	99.33	100.00
Last Mean (%)	96.67	97.71	97.75	98.02	97.89	98.38	98.83

## Data Availability

The CWRU dataset is available at https://engineering.case.edu/bearingdatacenter/download-data-file (accessed on 6 November 2024). The XJTU-SY dataset is available at https://biaowang.tech/xjtu-sy-bearing-datasets (accessed on 6 November 2024).
